# [Retracted] 5‑Fluorouracil‑induced apoptosis in colorectal cancer cells is caspase‑9‑dependent and mediated by activation of protein kinase C‑δ

**DOI:** 10.3892/ol.2025.15042

**Published:** 2025-04-14

**Authors:** Nizar M. Mhaidat, Mohammed Bouklihacene, Rick F. Thorne

Oncol Lett 8: 699–704, 2014; DOI: 10.3892/ol.2014.2211

Following the publication of the above paper, concerns were raised about the presentation of data in Figs. 6 and 9. In Fig. 6 (and Fig. 9, which shared the same control β-actin protein bands), it was drawn to the Editor's attention that the leftmost pair of the β-actin bands shown for the HCT116 experimental gels were strikingly similar to the rightmost β-actin bands featured for the SW480 gels; furthermore, in Fig. 9, the phosphorylated (P)-PKCε and PKCε panels for both the HCT116 and SW480 experiments also looked remarkably similar.

Upon investigating this matter independently in the Editorial Office, the Editor of *Oncology Letters* has decided that this paper should be retracted from the Journal on the grounds of a lack of confidence in the presented data, with probable errors having been made in compiling the data in these figures. The authors were asked for an explanation to account for these concerns, but the Editorial Office did not receive a reply. The Editor apologizes to the readership for any inconvenience caused.

